# Effect of High-Pressure Processing on the Physicochemical and Emulsifying Properties of Pea Protein Isolate

**DOI:** 10.3390/foods15183237

**Published:** 2026-09-13

**Authors:** Napassorn Salamun, Suteera Vatthanakul, Bootsrapa Leelawat, Witoon Prinyawiwatkul

**Affiliations:** 1 Department of Food Science and Technology, Faculty of Science and Technology, Thammasat University, Pathumthani 12121, Thailand; napassorn.sala@dome.tu.ac.th (N.S.); leelawat@tu.ac.th (B.L.); 2 Center of Excellence in Food Science and Innovation, Thammasat University, Pathumthani 12121, Thailand; 3 Agricultural Center, School of Nutrition and Food Sciences, Louisiana State University, Baton Rouge, LA 70803-4200, USA; wprinya@lsu.edu

**Keywords:** pea protein isolate, high-pressure processing, protein functional properties, emulsion properties

## Abstract

This study investigated the effects of high-pressure processing (HPP) on the physicochemical and functional properties of pea protein isolate (PPI). PPI was HPP-treated at 0.1 (control), 400, and 500 MPa, and its protein solubility, oil-holding capacity (OHC), surface hydrophobicity (H_0_), ζ-potential, and emulsion droplet size (D_4,3_) were evaluated. Results showed that HPP significantly improved protein solubility, reaching 38.61% at 400 MPa compared with 12.45% in the untreated PPI powder. OHC and H_0_ of PPI also increased after HPP, suggesting its enhanced interaction with oil and exposure of hydrophobic residues. Emulsions stabilized with HPP-treated PPI exhibited smaller droplet sizes and higher absolute ζ-potential values, suggesting improved initial emulsifying properties. The enhancement in functional properties may be associated with changes in surface hydrophobicity and charge characteristics, potentially reflecting pressure-induced conformational rearrangements. However, a slight decrease in solubility and surface hydrophobicity was observed at 500 MPa, which may be associated with pressure-induced protein aggregation. Overall, HPP effectively modified PPI surface characteristics, leading to improved emulsifying and functional performance, highlighting its potential application in food formulations requiring good protein dispersibility and emulsifying properties.

## 1. Introduction

In recent years, interest in the development of plant-based food and beverage products has increased continuously, driven by growing consumer awareness of health, sustainability, and environmental impacts. In particular, plant-based protein products have gained widespread popularity as alternatives to animal-derived products. Various plant protein sources have been extensively studied and applied in the food industry due to their nutritional value and potential to support sustainable food systems [1]. Among these sources, pea protein has received considerable attention owing to its high protein content and a well-balanced essential amino acid profile, containing all nine essential amino acids. Pea protein is particularly rich in lysine, which contributes to immune function, and branched-chain amino acids (BCAAs), including leucine, isoleucine, and valine, which play important roles in promoting muscle growth. In addition, pea protein exhibits relatively good digestibility compared with some other plant proteins, making it suitable for incorporation into food and beverage products. Another notable advantage of pea protein is its low allergenic potential (hypoallergenic), especially when compared with soy protein, a widely used plant protein source that has been associated with allergic reactions in certain consumer groups [2,3]. Consequently, pea protein is considered a promising alternative for the development of food and beverage products targeting consumers with soy allergies or those seeking to limit the intake of animal-derived proteins. Despite its favorable nutritional value and safety, pea protein still exhibits limitations in functional properties, particularly in protein beverage applications. Common challenges include low solubility, limited gelation capacity, and suboptimal emulsifying properties. These limitations are closely associated with the molecular composition and compact structural organization of pea protein. Globulins constitute the major fraction of pea protein (approximately 65–85%), with 7S vicilin and 11S legumin representing the dominant storage proteins. The compact globular nature of these proteins restricts molecular flexibility and limits the exposure of surface-active functional groups, thereby constraining key functional properties such as solubility, emulsification, and gelation [4]. To overcome these challenges, various approaches have been investigated to improve the functional properties of pea protein, including pH adjustment, enzymatic treatment, thermal processing, ultrasonication, and the application of advanced processing technologies [5,6,7]. However, some of these methods may negatively affect nutritional quality or induce undesirable sensory changes. Therefore, the selection of processing technologies capable of effectively modifying protein functionality without compromising the fundamental protein structure has attracted increasing interest.

High-pressure processing (HPP) is a non-thermal food processing technology that has gained significant attention in recent years. The HPP process typically involves food products packaged in suitable containers, a pressure-transmitting medium (commonly water), and a hydrostatic pressure generation system. The application of HPP has demonstrated considerable potential in preserving various quality attributes of foods, including physical properties, aroma, color, and flavor, maintaining characteristics close to those of fresh products. Generally, HPP is conducted at pressures ranging from approximately 400 to 600 MPa (58,000–87,000 psi) [8]. Such high pressure can affect protein conformation at the secondary, tertiary, and quaternary levels without disrupting primary covalent bonds. These pressure-induced changes may enhance the exposure of functional groups, which are important for protein functionality [9]. While previous studies have investigated the effects of HPP on PPI and its functional properties, the reported effects vary depending on pressure level, treatment time, pH, and experimental conditions. For example, Hall and Moraru (2021) [10] compared HPP and heat treatment in pea proteins, while other studies investigated PPI at different pressure levels and pH conditions [11,12]. These studies differed in processing conditions and the properties evaluated, making direct comparison of the functional responses across studies difficult. Although several physicochemical and functional properties of PPI have been investigated previously, differences in experimental conditions make it difficult to establish how these properties respond collectively to HPP. Therefore, the present study evaluated protein solubility, surface hydrophobicity (H_0_), ζ-potential, oil-holding capacity (OHC), and initial emulsion droplet size (D_4,3_), following HPP treatment at 400 and 500 MPa. This approach provides additional information on how these physicochemical and functional properties respond collectively to HPP and their potential relevance to plant-based food and beverage products and other emulsion-based food systems.

Accordingly, the objective of this study was to investigate the effects of HPP on the physicochemical and functional properties of pea protein isolate. The study evaluated protein solubility, surface hydrophobicity (H_0_), ζ-potential, oil-holding capacity (OHC), and initial emulsion droplet size (D_4,3_) following HPP treatment at 400 and 500 MPa. The findings are expected to provide valuable insights into the potential of HPP for improving the functionality of pea protein isolate and to serve as a scientific basis for its potential application in plant-based food and beverage systems in the future.

## 2. Materials and Methods

### 2.1. Materials

Pea protein isolate (PPI; 82.03% protein) was purchased from Truly Organics Co., Ltd. (Chonburi, Thailand). Refined soybean oil was obtained from Thanakorn Vegetable Oil Products Co., Ltd. (Samut Prakan, Thailand). Bradford reagent was obtained from Tokyo Chemical Industry Co., Ltd. (Tokyo, Japan), and bovine serum albumin (BSA) was purchased from Thermo Fisher Scientific Inc. (Rockford, IL, USA). HPP was performed using a HPP unit (HPP600 MPa, BaoTou KeFa High Pressure Technology Co., Ltd., Baotou, China). All chemicals used in this study were of analytical grade.

### 2.2. Preparation of High-Pressure-Treated Pea Protein Isolate

Protein solutions were prepared for HPP following a modified method of Gül et al. [13]. Pea protein isolate (PPI) was dispersed in distilled water to obtain a concentration of 12% (*w*/*w*), yielding an initial pH of approximately 7.4 without pH adjustment. The protein solution was then transferred into polyethylene bags and hermetically sealed. HPP was conducted at 400 and 500 MPa for 10 min using a high-pressure system with water as the pressure-transmitting medium. The processing temperature was approximately 25 ± 2 °C (ambient temperature). The come-up time to reach the target pressures was approximately 30–40 s, and decompression was completed within 30 s. After the HPP treatment, the samples were freeze-dried to obtain HPP-treated PPI powder. The untreated PPI powder (0.1 MPa) underwent the same hydration, packaging, 10 min holding, and freeze-drying procedures as the HPP-treated samples, differing only in the absence of high-pressure treatment. All samples (0.1, 400, and 500 MPa) were pre-frozen from −10 °C to −40 °C and freeze-dried simultaneously using a vacuum freeze dryer (DL-0.5, Kinetic Engineering, Samut Prakan, Thailand). Sublimation and desorption were conducted under a system vacuum of 20.0 Pa (0.2 mbar), with the shelf temperature programmed from 0 °C to a final temperature of 40 °C for a total duration of 22.5 h to obtain untreated and HPP-treated PPI powders. For subsequent functional testing, the freeze-dried PPI powders were redispersed in distilled water, and the pH of the resulting dispersions was recorded as approximately 7.2–7.5. In this study, the HPP holding time was maintained at a fixed duration of 10 min for all treatments to standardize the processing conditions. While varying holding times were beyond the scope of this investigation, future research examining different holding times could provide further insights into their effects on protein structural modification.

### 2.3. Determination of Protein Solubility

Protein solubility was measured according to a modified method described by Queirós et al. [11]. PPI powder was dispersed in distilled water to obtain a concentration of 1% (*w*/*v*). The suspension was vigorously mixed using a vortex mixer for 5 min and allowed to stand for an additional 15 min at room temperature to ensure adequate hydration prior to centrifugation. Subsequently, the mixture was centrifuged at 12,000× *g* for 10 min. The protein content in the supernatant was determined using the Bradford assay. A bovine serum albumin (BSA) standard curve was prepared by diluting 1 mg/mL BSA stock solution to concentrations of 0, 120, 300, 480, 660, and 840 µg/mL, using distilled water as the blank. For the assay, 0.5 mL of either the sample supernatant or the BSA standard was mixed with 4 mL of Bradford reagent, vortexed, and allowed to react. The absorbance was measured at 595 nm within 30 min of reagent addition. Protein solubility was calculated using the following equation:

$$Protein\;solubility\left(\%\right)=\frac{C_{supernatant}}{C_{initial\;PPI}}\times 100$$

where C_supernatant_ is the protein concentration in the supernatant and C_initial PPI_ is the initial PPI concentration in the dispersion.

### 2.4. Determination of Oil-Holding Capacity (OHC)

OHC was determined using a modified method described by Gul et al. [14]. Briefly, 1 g of PPI powder was mixed with 10 mL of commercial soybean oil in a centrifuge tube. The mixture was vortexed thoroughly for 5 min and allowed to stand at room temperature (25 ± 2 °C) for 10 min to facilitate lipid–protein interactions. Subsequently, the suspension was centrifuged at 10,000× *g* for 15 min. The unbound oil supernatant was carefully decanted, and the tube was inverted and drained for 2 min to remove residual oil. The weight of the remaining residue was recorded, and OHC was calculated using the following equation:

$$\mathrm{OHC}\;(g\;\mathrm{oil}/g\;\mathrm{sample})=\frac{W_{sediment+oil}-W_{dry\;sample}}{W_{dry\;sample}}$$

where $W_{sediment+oil}$ is the weight of the sediment containing bound oil after centrifugation and removal of unbound oil, and $W_{dry\;sample}$ is the initial weight of the dry PPI sample.

### 2.5. Emulsion Preparation

Emulsions were prepared with modifications from the method of Khan et al. [15]. PPI powder was dispersed in distilled water at a concentration of 1% (*w*/*w*). A total of 7.5 g of the protein solution was mixed with 2.5 g of soybean oil, corresponding to an oil fraction of 25% (*w*/*w*). The mixture was homogenized using a high-speed homogenizer (X10/25, Ystral GmbH, Ballrechten-Dottingen, Germany) at 13,000 rpm for 2 min at room temperature to form an emulsion.

### 2.6. Determination of Zeta Potential of Emulsion Droplets

The zeta potential (ζ-potential) of emulsion droplets was determined according to the method of Tan et al. [16]. The freshly prepared emulsion was diluted 100-fold with distilled water (1:100, *v*/*v*) using the same lot of distilled water for all samples and immediately subjected to measurement. The measurements were performed at 25.0 °C, and the conductivity after dilution ranged from 0.430 to 0.435 mS/cm. The ζ-potential was then analyzed using a Zetasizer Nano ZS (Malvern Instruments Ltd., Worcestershire, UK) with 20 runs per measurement.

### 2.7. Determination of Emulsion Droplet Size (D_4,3_)

The particle size distribution of emulsion droplets was measured using a Mastersizer 3000 (Malvern Instruments Ltd., Worcestershire, UK). Scattering data were analyzed to determine the particle size distribution, which was reported as the volume-weighted mean diameter (D_4,3_). For the analysis, a particle refractive index of 1.520 was used based on the reported refractive index of pea protein [17], comparable to the RI of 1.510 previously applied to proteins/emulsions in laser-diffraction analysis of PPI and PPI emulsions [18]. A particle absorption index of 0.010 was also used. Distilled water was employed as the dispersant, with a dispersant refractive index of 1.330 [19].

### 2.8. Determination of Surface Hydrophobicity (H_0_)

H_0_ was determined according to the method of Tan et al. [16] with slight modifications. PPI powder was dispersed in 0.01 M phosphate buffer (pH 7.4) at 10% (*w*/*v*) in centrifuge tubes, followed by vortex mixing. The suspension was then centrifuged at 10,000× *g* for 15 min at 4 °C, and the supernatant was carefully collected for further analysis. The protein concentration of the supernatant was determined using a NanoDrop spectrophotometer (Thermo Fisher Scientific, Waltham, MA, USA). The solution was subsequently diluted with 0.01 M phosphate buffer (pH 7.4) to obtain five different concentrations (0.05–0.8 mg/mL). All samples were filtered through a 0.45 μm syringe filter to remove residual particles and minimize interference during fluorescence measurement. A total of 4 mL of each protein solution was mixed with 20 μL of 8 mM 8-anilinonaphthalene-1-sulfonic acid (ANS). The fluorescence intensity was measured using a multi-mode microplate reader (SpectraMax iD3, Molecular Devices, San Jose, CA, USA) at excitation and emission wavelengths of 395 nm and 475 nm, respectively. A plot of fluorescence intensity (*y*-axis) against protein concentration (*x*-axis) was constructed, and the slope of the linear regression was used to determine the H_0_ of the protein.

### 2.9. Statistical Analysis

The experiment was conducted using a completely randomized design (CRD). Two independent biological replicates (*n* = 2) were prepared, with each measured in triplicate. To avoid pseudo-replication, statistical analysis was performed on the biological means. Analysis of variance (ANOVA) and Duncan’s New Multiple Range Test were conducted using IBM SPSS Statistics (version 29.0.1.0) to determine differences at *p* < 0.05. Using two biological replicates is acknowledged as a limitation of this study; therefore, future research with ≥3 biological replicates is recommended to further validate these findings.

## 3. Results and Discussion

### 3.1. Protein Solubility

The protein solubility of PPI treated with HPP at 400 and 500 MPa was compared with that of the untreated PPI powder (0.1 MPa). The results showed that HPP significantly improved the solubility of PPI. Similar findings were reported in melon seed protein isolate and pumpkin seed protein isolate treated with HPP [20,21]. This pressure-induced enhancement in solubility is particularly advantageous for food matrix design, as higher soluble protein concentrations may facilitate greater adsorption of proteins at oil–water or air–water interfaces and potentially contribute to improved emulsifying properties [22].

**Figure 1:**
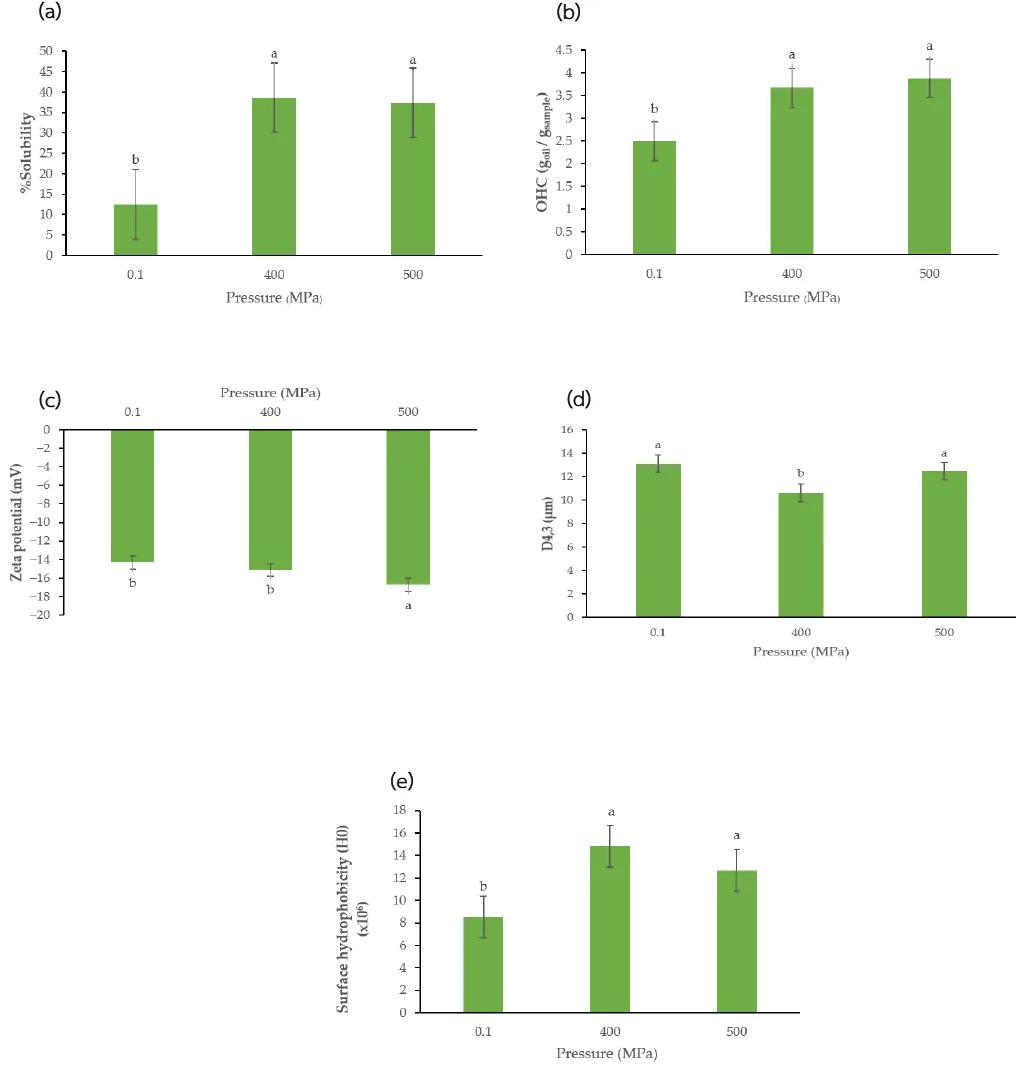
Effect of high-pressure processing (0.1, 400, and 500 MPa) on the functional and physicochemical properties of PPI powder: (**a**) % solubility (F = 744.065, *p* < 0.001), (**b**) oil-holding capacity, OHC (F = 18.579, *p* = 0.02), (**c**) zeta potential of emulsion (F = 23.494, *p* = 0.015), (**d**) emulsion droplet size, D_4,3_ (F = 13.196, *p* = 0.033), and (**e**) surface hydrophobicity, H_0_ (F = 12.230, *p* = 0.036). Values are presented as mean ± standard deviation from two independent biological replicates. Significant differences among treatment groups were determined by one-way ANOVA followed by Duncan’s New Multiple Range Test (*p* < 0.05); different letters indicate significant differences among treatments.

The solubility of PPI reached 38.61 ± 0.69% at 400 MPa and 37.36 ± 0.34% at 500 MPa, whereas the control sample exhibited a solubility of only 12.45 ± 1.08% (Figure 1a). This indicates that HPP-treated PPI showed approximately threefold higher solubility compared with the untreated PPI powder. PPI typically exhibits limited water solubility due to its compact and highly ordered molecular structure, which promotes protein aggregation and the formation of insoluble particles, thereby restricting its functional properties [5]. However, in the present study, protein solubility increased after HPP treatment, reaching the highest value at 400 MPa. A slight decrease was observed at 500 MPa, although the value was not significantly different from that at 400 MPa.

The increase in protein solubility at a moderate pressure level (400 MPa in this study) may be associated with changes in protein conformation and surface characteristics following HPP treatment. High hydrostatic pressure may affect protein conformation through changes in non-covalent interactions, such as hydrogen bonds, hydrophobic interactions, and electrostatic interactions, potentially altering protein surface characteristics and the exposure of hydrophobic and polar groups. Such changes may enhance protein–water interactions and contribute to improved protein dispersion in aqueous systems. In addition, possible dissociation of insoluble protein complexes may further facilitate protein hydration and contribute to the improvement of solubility [13]. At higher pressure levels (500 MPa in this study), however, greater exposure of hydrophobic groups may favor protein–protein interactions and potentially contribute to aggregate formation, which could result in a slight decrease in protein solubility. A similar trend was reported in walnut protein isolate, where solubility decreased as the pressure level increased due to the formation of high-molecular-weight aggregates [23]. This phenomenon may explain the slight reduction in solubility observed at 500 MPa in the present study (Figure 1a).

### 3.2. Oil-Holding Capacity (OHC)

High-pressure processing (HPP) significantly improved the OHC of PPI powder (*p* < 0.05), which may enhance oil-binding functionality and potentially contribute to aroma retention and sensory properties in food systems [24]. The OHC of PPI increased after HPP treatment. The untreated PPI powder (0.1 MPa) showed an OHC of 2.50 ± 0.04 g oil/g sample, whereas the values increased to 3.67 ± 0.25 and 3.88 ± 0.34 g oil/g sample at 400 and 500 MPa, respectively (Figure 1b).

These results suggest that HPP treatment enhanced the oil retention ability of the protein. The increase in OHC may be associated with changes in protein surface characteristics and greater exposure of hydrophobic sites, which could facilitate interactions with oil molecules [25]. Similar results were reported in previous studies on peanut protein isolate by He et al. [26], where HPP treatment significantly increased the oil-binding capacity of the proteins due to pressure-induced structural changes.

### 3.3. ζ-Potential of Emulsion

In the present study, the ζ-potential of emulsions stabilized with HPP-treated PPI was more negative than that of the untreated PPI powder (Figure 1c). The emulsion prepared with pea protein isolate treated at 500 MPa showed the highest absolute ζ-potential value (−16.7 ± 0.1 mV), followed by the sample treated at 400 MPa (−15.1 ± 0.2 mV), while the untreated PPI powder exhibited a ζ-potential value of −14.3 ± 0.5 mV (Figure 1c). These results indicate that the absolute ζ-potential values increased with increasing pressure levels. This shift may be associated with greater exposure of charged groups on the protein surface following HPP treatment, which could increase electrostatic repulsion [27]. Furthermore, a higher absolute ζ-potential generally promotes electrostatic repulsion between droplets, which may reduce droplet aggregation and contribute to the initial physical stability of colloidal systems [28,29]. Moreover, high zeta potential values do not necessarily represent high colloidal stability in dispersed systems, as stability may also be influenced by other factors such as particle size and interfacial properties [30]. The effect of HPP treatment on emulsion droplet size and its implications for initial emulsifying properties will be discussed next.

### 3.4. Emulsion Droplet Size (D_4,3_)

In this study, emulsions stabilized with HPP-treated PPI exhibited smaller droplet sizes than the untreated PPI powder. The emulsion prepared with PPI treated at 400 MPa exhibited the smallest droplet size (10.6 ± 0.5 μm), which was significantly lower than that of the untreated PPI powder (13.1 ± 0.5 μm). The sample treated at 500 MPa showed a slightly larger droplet size (12.5 ± 0.3 μm) compared with the 400 MPa treatment, but it remained smaller than that of the untreated PPI powder (Figure 1d).

HPP treatment may alter protein conformation and surface characteristics, potentially increasing the exposure of hydrophobic regions [13,31]. This enhanced exposure of hydrophobic sites on the protein surface can promote protein adsorption at the oil–water interface, thereby facilitating the formation of smaller emulsion droplets [32]. However, excessive pressure may also promote protein–protein interactions due to the increased exposure of hydrophobic and sulfhydryl groups, which can lead to protein aggregation [33]. Such aggregation may reduce the ability of proteins to effectively adsorb at the oil–water interface, thereby resulting in slightly larger droplet sizes at higher pressure levels as observed at 500 MPa in this study (Figure 1d). Consequently, finer oil droplet sizes are generally associated with improved emulsion quality and may contribute to greater physical stability [12], and the smaller droplet size achieved at 400 MPa corresponds to a greater specific surface area of the dispersed phase, which may be favorable for the initial emulsifying properties of the emulsion. It should be noted that the D_4,3_ and ζ-potential values evaluated in this study reflect the initial physical state of freshly prepared emulsions; long-term storage stability requires further investigation.

### 3.5. Surface Hydrophobicity (H_0_)

The results indicated that PPI treated with HPP at 400 and 500 MPa exhibited significantly higher H_0_ values compared with the untreated PPI powder (Figure 1e). The increase in H_0_ may indicate greater exposure of hydrophobic regions on the protein surface following HPP treatment, consistent with the role of H_0_ as an indicator of surface-exposed hydrophobic groups and changes in protein tertiary structure [34,35]. This increase could reflect changes in protein conformation and surface accessibility at different pressure levels [25]. However, a slight decrease in H_0_ was observed at 500 MPa compared with 400 MPa. This reduction may be related to protein aggregation at higher pressure levels, which can lead to the re-association of protein molecules and a decrease in the exposure of hydrophobic groups on the protein surface [36]. The increased H_0_ values were consistent with the observed increase in protein solubility (Figure 1a) and the reduction in emulsion droplet size (Figure 1d). This was quantitatively supported by Pearson correlation analysis, which showed strong positive correlations of solubility with both H_0_ (r = 0.898, *p* = 0.015) and OHC (r = 0.947, *p* = 0.004), as well as a negative correlation between emulsion droplet size (D_4,3_) and H_0_ (r = −0.795, *p* = 0.059). These findings suggest that changes in protein surface characteristics following HPP treatment were associated with changes in protein functionality under the conditions investigated. The positive correlation between solubility and H_0_ suggests that greater exposure of hydrophobic groups was associated with higher protein solubility under the conditions investigated. In addition, the negative correlation between H_0_ and emulsion droplet size suggests that increased surface hydrophobicity may have contributed to more effective protein adsorption at the oil–water interface, resulting in smaller droplets. Similar findings have also been reported for pea protein isolates, where enhanced H_0_ was associated with improved solubility and the formation of smaller emulsion droplets [37,38]. The improved overall physical stability of the dispersion may be attributed to a combination of stabilizing factors, such as smaller particle size, and variations in surface attributes, including the altered zeta potential [39,40]. In addition, surface charge can influence the distribution of surrounding ions, which may affect particle mobility in solutions and consequently impact zeta potential [40].

## 4. Conclusions

This study demonstrated that high-pressure processing (HPP) effectively improved some functional and physicochemical properties of pea protein isolate (PPI). Treatment at 400 and 500 MPa significantly enhanced protein solubility, OHC, and H_0_, while reducing emulsion droplet size and increasing the absolute ζ-potential of emulsions, suggesting potentially improved initial dispersion stability. The observed improvements may be associated with changes in surface characteristics, including the exposure of hydrophobic and charged groups, which may influence protein–water and protein–oil interactions. Although a slight decrease in solubility and surface hydrophobicity was observed at 500 MPa, which may be associated with protein aggregation at the higher pressure, the overall effect of HPP was positive. These findings highlight the potential of HPP as a non-thermal technique to modify PPI surface characteristics and enhance emulsifying performance. The enhanced functional properties of PPI following HPP treatment suggest its potential applicability in food formulations requiring protein-based emulsions with desirable initial emulsifying properties, such as beverages, dressings, and plant-based products. These findings may also support the potential use of HPP-treated PPI as a functional protein ingredient in aqueous and emulsion-based food systems where protein solubility and emulsifying properties are important for maintaining homogeneous dispersions. Further evaluation of HPP-treated PPI in more complex food matrices, including storage stability, phase separation, rheological properties, and sensory characteristics, would also help determine its practical applicability in food systems. Future studies exploring a wider range of pressure levels and holding times, together with direct structural analyses, could provide further insights into the structural changes and aggregation behavior of PPI. Overall, this study provides valuable insight into the functional responses of PPI to HPP treatment and would contribute to the development of high-quality protein-enriched food systems.

## Data Availability

The original contributions presented in the study are included in the article, further inquiries can be directed to the corresponding author.
